# Retraction: miR-150-5p inhibits hepatoma cell migration and invasion by targeting MMP14

**DOI:** 10.1371/journal.pone.0338550

**Published:** 2025-12-17

**Authors:** The PLOS One Editors

After this article [1] was published, concerns were raised about Figs 2 and 3.

Specifically,

- In all panels in Figs 2B and 2C:
  ◦ Similarities were noted between multiple regions within each panel.
  ◦ Several areas around groups of cells appear to be discontinuous with adjacent background areas.
- In Fig 3C, both Actin bands of the Huh7 cell line appear to have horizontal discontinuities above and below the bands.

In light of the nature and extent of the above concerns which question the reliability and integrity of the results presented in [1], the *PLOS One* Editors retract this article.

All authors either did not respond directly or could not be reached.
